# Pan-cancer analysis suggests histocompatibility minor 13 is an unfavorable prognostic biomarker promoting cell proliferation, migration, and invasion in hepatocellular carcinoma

**DOI:** 10.3389/fphar.2022.950156

**Published:** 2022-08-15

**Authors:** Jun Liu, Wenli Li, Liangyin Wu

**Affiliations:** ^1^ Department of Clinical Laboratory, Yue Bei People’s Hospital, Shantou University Medical College, Shaoguan, Guangdong, China; ^2^ Medical Research Center, Yue Bei People’s Hospital, Shantou University Medical College, Shaoguan, Guangdong, China; ^3^ Reproductive Medicine Center, Yue Bei People’s Hospital, Shantou University Medical College, Shaoguan, Guangdong, China

**Keywords:** histocompatibility minor 13, pan-cancer, prognostic biomarker, drug sensitivity, cell proliferation

## Abstract

Histocompatibility Minor 13 (HM13) encoding the signal peptide peptidase plays an important role in maintaining protein homeostasis but its role in tumors remains unclear. In this study, 33 tumor RNA-seq datasets were extracted from The Cancer Genome Atlas (TCGA) database, and the pan-cancer expression profile of HM13 was evaluated in combination with The Genotype-Tissue Expression (GTEx) datasets. The prognostic significance of abnormal HM13 pan-cancer expression was evaluated by univariate Cox regression and Kaplan-Meier analyses. Co-expression analysis was performed to examine the correlation between abnormal pan-cancer expression of HM13 and immune cell infiltration, immune checkpoint, molecules related to RNA modification, tumor mutational burden (TMB), microsatellite instability (MSI), and other related molecules. CellMiner database was used to evaluate the relationship between the expression of HM13 and drug sensitivity. The results showed overexpression of HM13 in almost all tumors except kidney chromophobe (KICH). Abnormally high expression of HM13 in adrenocortical carcinoma (ACC), kidney renal papillary cell carcinoma (KIRP), uveal melanoma (UVM), liver hepatocellular carcinoma (LIHC), brain lower grade glioma (LGG), head and neck squamous cell carcinoma (HNSC), and kidney renal clear cell carcinoma (KIRC) was associated with poor prognosis. Expression of HM13 correlated strongly with pan-cancer immune checkpoint gene expression and immune cell infiltration. Drug sensitivity analysis indicated that the expression of HM13 was an excellent predictor of drug sensitivity. We verified that both mRNA and protein levels of HM13 were abnormally upregulated in HCC tissues, and were independent risk factors for poor prognosis. Furthermore, interference with HM13 expression in Huh-7 and HCCLM3 cells significantly inhibited proliferation, migration, and invasion. Therefore, our findings demonstrate that HM13 is a potential pan-cancer prognostic marker, thus providing a new dimension for understanding tumor development.

## Introduction

Cancer is a grave threat to public health worldwide owing to the high morbidity and mortality (Wu et al., 2019). In recent years, the overall death rate due to cancer in developed countries such as the United States has decreased owing to advanced medical technology and the concept of precise and individualized medicine, however, the situation remains unfavorable (Golemis et al., 2018). In the United States, daily, 1,700 people die of cancer (Delman, 2020). Incidence and mortality of cancer in developing countries are showing an increasing trend. With the rise in the aging population globally, researchers predict that the incidence of cancer will double by 2070 relative to 2020 (Soerjomataram and Bray, 2021). In recent years, although the application of immunotherapy and individualized targeted therapy has been successful to an extent, the rate of effectiveness is only 20%, and that of survival among patients remains unsatisfactory. Therefore, the identification of new tumor markers is necessary to facilitate early diagnosis and enhance the prognostic assessment of cancer, thereby increasing the overall survival rate.

The signal peptide peptidase encoded by Histocompatibility Minor 13 (HM13) is localized to the endoplasmic reticulum and is mainly implicated in the regulation of the US2 pathway, which in turn is responsible for the cleavage of the pro-protease and catalytic hydrolysis of proteins in the membrane (Loureiro et al., 2006). Previous studies suggest that HM13 is crucial in the regulation of the production of lymphocyte surface (HLA-E) epitopes that generate MHCI-like signal peptides recognized by the immune system (Lemberg et al., 2001). Moreover, accumulating evidence shows that HM13 is involved in cell signaling and intracellular communication (Brown et al., 2000; Urban et al., 2001). Jian Zhou et al. show that HM13 is highly expressed in lung cancer tissues and is a potential marker for early diagnosis of lung cancer (Zhou et al., 2021). Tine Goovaerts et al. demonstrate that methylation at the promoter level in HM13 is abnormally dysregulated in breast cancer, thus leading to its aberrantly high expression and underlies its involvement (Goovaerts et al., 2018). Currently, very little research has disclosed how HM13 plays a role in tumor development. Further, its pan-cancer expression and role in tumorigenesis remain unclear.

In recent years, with advancements in -omics technology and the application of high-throughput sequencing methods, large-omics data have been generated, which are expected to facilitate studies on the occurrence and related mechanisms underlying diseases (Sherman and Salzberg, 2020). Thus, in this study, we analyzed the pan-cancer expression of HM13 in tissues using RNA-seq datasets in The Cancer Genome Atlas (TCGA). Relying on the large clinical follow-up data, we investigated the effects of abnormal HM13 expression on overall and disease-free survival. Moreover, we elucidated the potential mechanism underlying HM13 action in tumor development and genesis and identified the correlation of HM13 expression with tumor mutation load, immune cell invasion, microsatellite instability, and tumor purity in the tumor microenvironment. Additionally, by transcriptomics and proteomics, we verified the expression and prognostic value of HM13 in hepatocellular carcinoma (HCC). Furthermore, the effects of HM13 were examined on cellular migration, proliferation, and invasion in HCC lines. Herein, the pan-cancer expression of HM13 in tissues was described using multi-omics data, thus providing a new dimension for understanding the occurrence of tumors.

## Materials and methods

### Acquisition of transcriptomic and clinical data

The unified standardized TCGA pan-cancer dataset and RNA-seq data from TCGA and GTEx in TPM format uniformly processed by the toil method were downloaded from the UCSC database (https://xenabrowser.net/). All relevant survival data from clinical follow-up were obtained. Transcriptome data of HCC (ICGC-LIRI-JP) and corresponding clinical data were extracted from the ICGC database (https://dcc.icgc.org/). Protein data of HCC (PDC000198) processed by Z-score standardization and the corresponding follow-up information were obtained from the CPTAC database (https://pdc.cancer.gov/). In addition, we obtained the microarray data and RNA-Seq of HCC from Gene Expression Omnibus (GEO) database, including GSE36376, GSE63898, GSE64041 and GSE202853 cohort. Microsatellite instability scores for 33 tumors were obtained from the literature review (Bonneville et al., 2017). Level 4 Simple Nucleotide Variation dataset for all TCGA samples processed using the MuTect2 software was obtained from TCGA (https://portal.gdc.cancer.gov/), and subsequently, the tumor mutation burden was calculated (Beroukhim et al., 2010). Purity and ploidy data for several tumors were obtained from the literature (Thorsson et al., 2018).

### Differential analysis and prognostic significance of HM13 expression

The differential expression of HM13 between normal and tumor tissues was analyzed by the Wilcoxon rank-sum test. The “survminer” and “survival” packages were used for Kaplan-Meier and univariate COX analyses to examine the effects of HM13 expression on the progression-free survival and overall survival; to estimate the statistical significance of the results, the log-rank test was employed.

### Correlation and functional enrichment analyses

Tumour Immune Estimation Resource is a website (TIMER, https://cistrome.shinyapps.io/timer/) used for comprehensively evaluating the levels of tumor immune cells infiltration, and was used to obtain information on six types of immune cell invasion in common tumors in TCGA. The corr. test function in the psych package (R software, version 2.1.6) was employed to calculate the Pearson’s correlation coefficient between pan-cancer HM13 expression and immune cell infiltration score. Additionally, we also calculated the Pearson’s correlation coefficient between HM13 expression and the previously reported immune checkpoint genes, RNA modified genes, TMB, MSI, purity, and ploidy in tumors. The functions of the GSVA package were used to perform ssGSEA analysis to predict the infiltration of 24 types of immune cells in HCC and calculate the association between immune cell infiltration levels and the HM13 expression (Bindea et al., 2013). The clusterProfiler package in R was used for GO annotation and KEGG pathway enrichment analyses. To construct the potential interaction network for HM13, the GeneMANIA database (http://genemania.org/) was used.

### Drug sensitivity analysis of HM13

CellMiner database integrates the correlation between drug sensitivity of tumor cells and genome data for tumor treatment (Reinhold et al., 2012). The CellMiner database was used to evaluate HM13 expression and drug sensitivity data. The Genomics of Drug Sensitivity in Cancer (GDSC) database (https://www.cancerrxgene.org/) was used to obtain drug response data for 265 compounds in 1,001 cancer cell lines. Additionally, the cell lines were categorized as high expression and low expression based on the median HM13 expression. The sensitivity of both cell lines to drugs was compared.

### Cell culture

Human normal hepatocytes, LO2, and the HCC cell lines, including HCCLM3, Huh7, and HepG2, were obtained from American Type Culture Collection (ATCC, Manassas, VA, United States). Huh7 and HCCLM3 cells were grown in DMEM supplemented with 10% fetal bovine serum (FBS). HepG2 cells were grown in MEM supplemented with 10% FBS. LO2 cells were grown in RPMI-1640 supplemented with 10% FBS. All cells were cultured in an incubator at 37°C with 5% CO_2_. Transfection were performed with 70%–80% cell density, according to the instructions of Lipo3000 (L3000015, Invitrogen, United States). ShRNA was expressed in pRNA-H1.1, and the sequences of shRNA-HM13 were as follows: shRNA-1, GCU​GGA​GAA​GAA​AGA​GAA​ATT​UUU​CUC​UUU​CUU​CUC​CAG​CTT; shRNA-2, GGC​UGG​AGA​AGA​AAG​AGA​ATT​UUC​UCU​UUC​UUC​UCC​AGC​CTT; shRNA-3, UGA​CAG​AGA​UGU​UCA​GUU​ATT​UAA​CUG​AAC​AUC​UCU​GUC​ATT.

### Quantitative reverse transcription-polymerase chain reaction

Total RNA in cells was extracted using the TRIpure reagent (RP1001, BioTeke, Beijing, China). The concentration of RNA in each sample was measured on an ultraviolet spectrophotometer (NANO 2000; Thermo, United States). The RNA was reverse transcribed into cDNA (D7160L, Beyotime, Shanghai, China) and stored at −80°C till further use. The primer sequences of HM13 were 5′-AGC​CTG​CCC​TCC​TAT​ACC​T-3′ and 3′-TGT​TCC​CTC​TTT​GGA​TTC​TG-5′, and the primer sequences of *β*-actin were 5′-GGC​ACC​CAG​CAC​AAT​GAA-3′, 3′-TAG​AAG​CAT​TTG​CGG​TGG-5'. Subsequently, a real-time polymerase chain reaction (RT-PCR) quantitative fluorescence analysis was conducted for four replicates. The RT-PCR reaction conditions were as follows: cDNA template 1μL, the primers (10 μM) 0.5 μl, SYBR GREEN mastermix (PC1150, Solarbio, Beijing, China) 10 μl, and the volumes were adjusted to 20 μl with dd H_2_O. The results were standardized against the gene expression of *β*-actin, and the relative levels of expression were estimated by the 2^−ΔΔ^ Ct method.

### Western blot analysis

After the cell confluency reached 90%, protein extraction, protein quantitative analysis, SDS-PAGE (WLA025, wanleibio, China) transferring onto PVDF membrane (IPVH00010, Millipore, United States), blocking with skim milk (Q/NYLB0039S, YiLi, China), incubation with primary antibody (20416-1-AP, Proteintech, China), and subsequently, secondary antibody (WLA023, wanleibio, China) was performed. Finally, the results were observed by luminescence using the ECL substrate (WLA003, wanleibio, China). The Gel image processing system (Gel-Pro-Analyzer software) (WD-9413B, LIUYI, China) was used to analyze the optical density values of the target bands.

### CCK8 assay

When the confluency of Huh7 and HCCLM3 cells reached 90%, these were collected and seeded into 96-well culture plates with 3×10^3^ cells in each well. The experiment was classified into three, namely, the no-operation, the empty vector, and the sh-HM13 groups. Each group was designed with five replicates. The CCK-8 assay was performed at 0, 24, 48, 72, and 96 h.

### Clone formation assay

Cells in each group were collected and inoculated in Petri dishes at the amount of 400 cells per dish. After 14 days, visible clones formed. The cells were washed 2 times with PBS after fixation of cells with paraformaldehyde (C104188, Aladdin, China). Giemsa R1 solution (D010, Nanjing Jiancheng, China) was added in the plate to stain for 1 min. We then washed the plate three times with water after satisfactory staining with R2 solution.

### Wound healing assay

The cells were collected, counted, and inoculated in a six-well plate. After adhering, the cells were transfected; 48 h following transfection, the original medium was replaced with the serum-free medium containing 1 μg/ml mitomycin C, and 1 hour later, the scratch test was performed using a 200 μl pipette tip. Subsequently, cells were photographed at 24 and 48 h.

### Transwell migration and invasion assay

The transwell chambers were constructed using Matrigel glue (356,234, Corning, United States). The cells were grown in six-well plates. When the confluency reached 90%, the cells were digested and diluted to a density of 5×10^4^ cells/well with serum-free medium. The invasion assay was performed as follows: Cells in the Transwell containing Matrigel glue were seeded in the upper chamber of 24-well plates, and 800 μl of the medium supplemented with 10% FBS was added to the lower chamber. The migration assay was performed as follows: In 24-well plates, the coated lower chambers of Transwell system were placed with medium containing 10% FBS in 800 μl. Upper chambers were filled with a 200 μl cell suspension. PBS washed the Transwell and 4% paraformaldehyde phosphate buffer was applied (20 min at room temperature), and stained with 0.5% crystal violet (5 min). Cells counting were performed 24 h later.

### Statistical analyses

GraphPad Prism 7.0 and R software (version 4.1.1) were used for calculations, graph plotting, and statistical analyses of all data in this study. For comparison between two groups of continuous variables, the statistical significance for the normal distribution was calculated by independent Student t-test, while differences between the non-normally distributed variables were estimated by Mann-Whitney *U*-test (Wilcoxon rank-sum test). All statistical *p*-values were two-tailed, with *p* < 0.05 indicating significance.

## Results

### Pan-cancer expression of HM13

**FIGURE 1:**
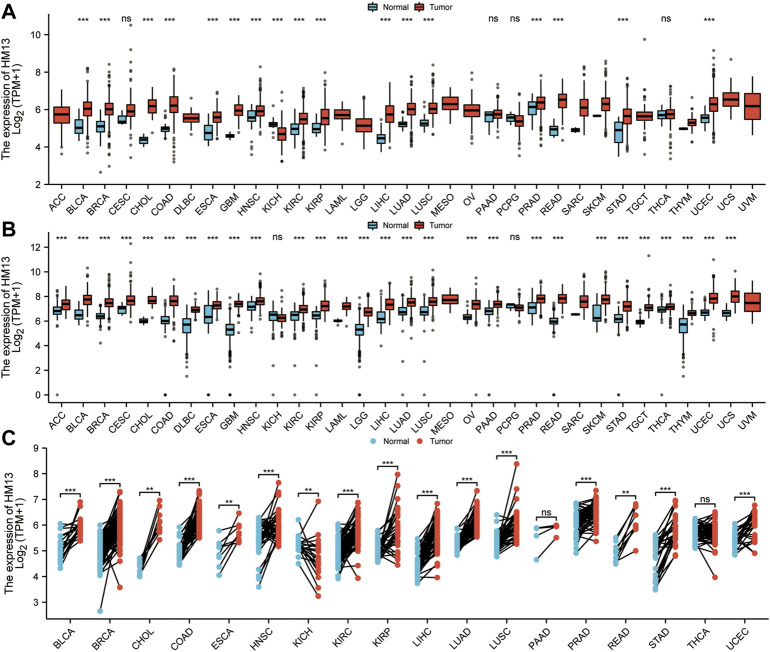
Differential expression analysis of HM13. **(A)**. The mRNA Expression level of HM13 from the TCGA database. **(B)**. The mRNA expression of HM13 from TCGA and GTEx databases. **(C)**. The expression differences of HM13 in tumor and corresponding adjacent tissues were compared with paired analysis. Mann-Whitney *U* test was used for this analysis, ns, *p* ≥ 0.05; *, *p* < 0.05; **, *p* < 0.01; ***, *p* < 0.001. ACC: Adrenocortical carcinoma, BLCA: Bladder urothelial carcinoma, BRCA: Breast invasive carcinoma, CESC: Cervical and endocervical cancers, CHOL: Cholangiocarcinoma, COAD: Colon adenocarcinoma, DLBC: Lymphoid Neoplasm Diffuse Large B-cell Lymphoma, ESCA: Esophageal carcinoma, GBM: Glioblastoma multiforme, HNSC: Head and Neck squamous cell carcinoma, KICH: Kidney Chromophobe, KIRC: Kidney renal clear cell carcinoma, KIRP: Kidney renal papillary cell carcinoma, LAML: Acute Myeloid Leukemia, LGG: Brain Lower Grade Glioma, LIHC: Liver hepatocellular carcinoma, LUAD: Lung adenocarcinoma, LUSC: Lung squamous cell carcinoma, MESO: Mesothelioma, OV: Ovarian serous cystadenocarcinoma, PAAD: Pancreatic adenocarcinoma, PCPG: Pheochromocytoma and Paraganglioma, PRAD: Prostate adenocarcinoma, READ: Rectum adenocarcinoma, SARC: Sarcoma, SKCM: Skin Cutaneous Melanoma, STAD: Stomach adenocarcinoma, STES: Stomach and Esophageal carcinoma, TGCT: Testicular Germ Cell Tumors, THCA: Thyroid carcinoma, THYM: Thymoma, UCEC: Uterine Corpus Endometrial Carcinoma, UCS: Uterine Carcinosarcoma, UVM: Uveal Melanoma.

RNA-seq data from TCGA showed that HM13 mRNA expression was up-regulated in several tumor tissues, including uterine corpus endometrial carcinoma (UCEC), cholangio carcinoma (CHOL), glioblastoma multiforme (GBM), breast invasive carcinoma (BRCA), KIRC, HNSC, LIHC, KIRP, lung squamous cell carcinoma (LUSC), lung adenocarcinoma (LUAD), bladder urothelial carcinoma (BLCA), colon adenocarcinoma (COAD), esophageal carcinoma (ESCA), prostate adenocarcinoma (PRAD), stomach adenocarcinoma (STAD), and rectum adenocarcinoma (READ) (Figure 1A). However, its level of expression in tumor tissues from patients with KICH was lower relative to the normal tissues (Figure 1A). Since TCGA database contains fewer normal samples, we combined the standardized GTEx and TCGA data from the UCSC database. Similarly, HM13 expression tended to be high in all tumor types (Figure 1B). Furthermore, paired analysis was performed to determine HM13 expression in tumors, and the results showed high HM13expression in several tumor tissues, including UCEC, CHOL, BRCA, KIRC, HNSC, LIHC, KIRP, LUSC, LUAD, BLCA, COAD, ESCA, PRAD, STAD, and READ (Figure 1C).

### Pan-cancer prognostic value of HM13 expression

**FIGURE 2:**
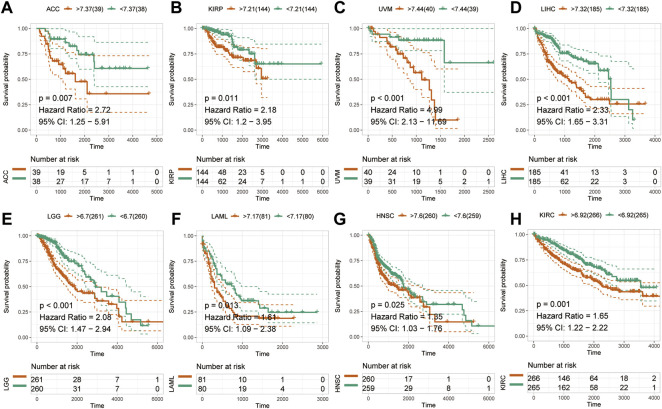
Effects of pan-cancer HM13 expression on overall survival. Kaplan-Meier method was used to evaluate the effects of abnormal expression of HM13 on the overall survival rate in ACC **(A)**, KIRP **(B)**, UVM **(C)**, LIHC **(D)**, LGG **(E)**, LAML **(F)**, HNSC **(G)**, and KIRC **(H)**. The *p*-value was calculated using the log-rank test.

**FIGURE 3:**
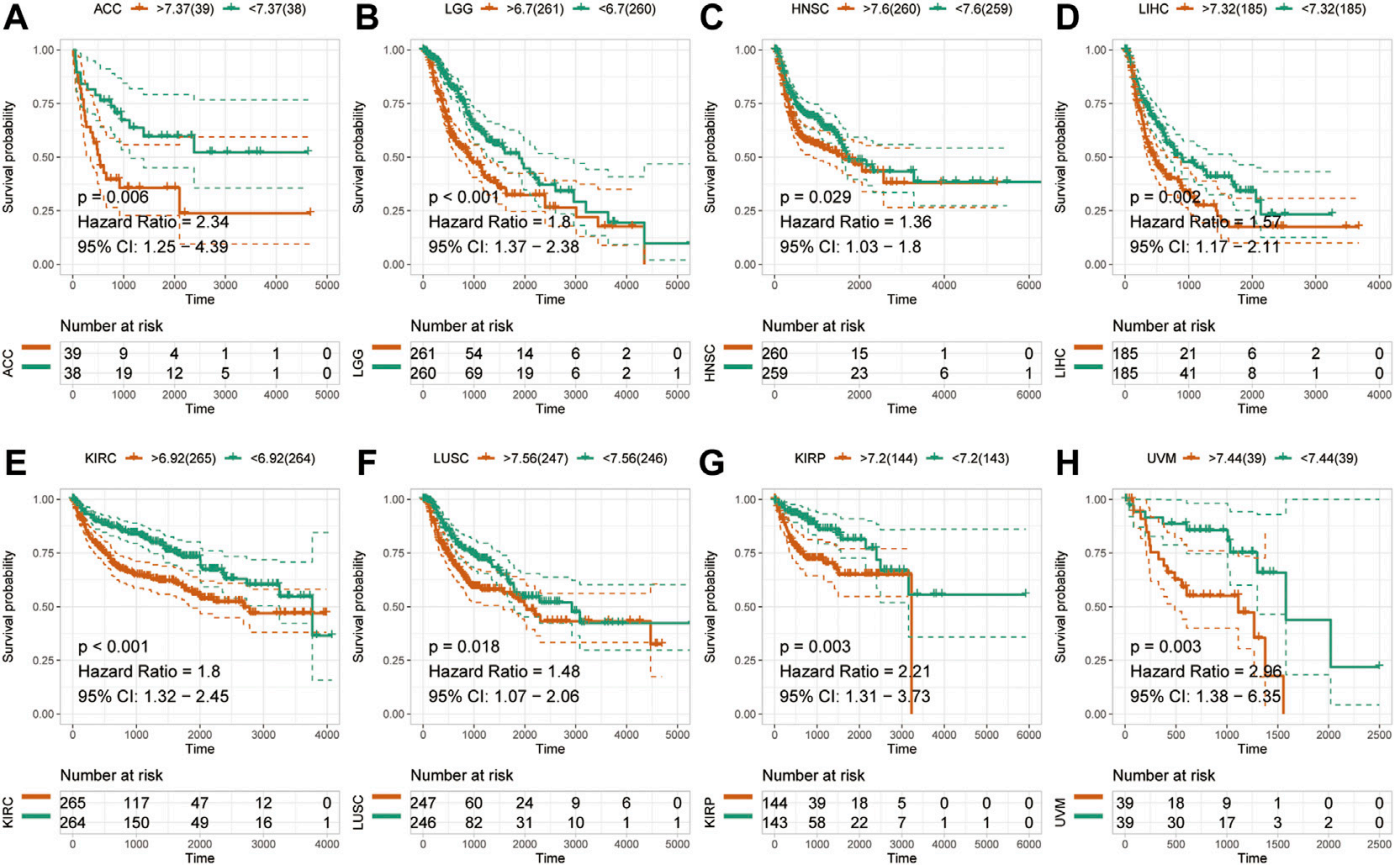
Effects of pan-cancer HM13 expression on disease-free survival. Kaplan-Meier method was used to evaluate the effects of abnormal expression of HM13 on the disease-free survival rate in ACC **(A)**, LGG **(B)**, HNSC **(C)**, LIHC **(D)**, KIRC **(E)**, LUSC **(F)**, KIRP **(G)**, and UVM **(H)**. The *p*-value was calculated using the log-rank test.

To examine the effect of abnormal pan-cancer HM13 expression on the overall survival time, a univariate Cox regression analysis was conducted. The results showed that in several tumors, abnormally high HM13 expression predicted worse overall survival time, including the ACC, BRCA, CESC, KIRC, KIRP, LAML, LGG, LIHC, LUAD, READ and UVM (Supplementary Figure S1). Next, Kaplan-Meier survival analysis was performed and the results showed that abnormally high expression of HM13 was associated with shorter overall survival time in ACC, KIRP, UVM, LIHC, LGG, LAML, HNSC and KIRC (Figures 2A–H). Next, the influence of HM13 expression on disease-free survival was investigated, and results of the univariate Cox analysis suggested that patients with ACC, CESC, KIRC, KIRP, LGG, LIHC, TGCG, and UVM showing high expression of HM13 had worse disease-free survival (Supplementary Figure S2). Kaplan-Meier analysis showed that abnormally high expression of HM13 correlated significantly with shorter disease-free survival time in ACC, LGG, HNSC, LIHC, KIRC, LUSC, KIRP, and UVM (Figures 3A–H).

### Correlation analysis for HM13 expression with levels of immune infiltration and RNA modification-related molecules

**FIGURE 4:**
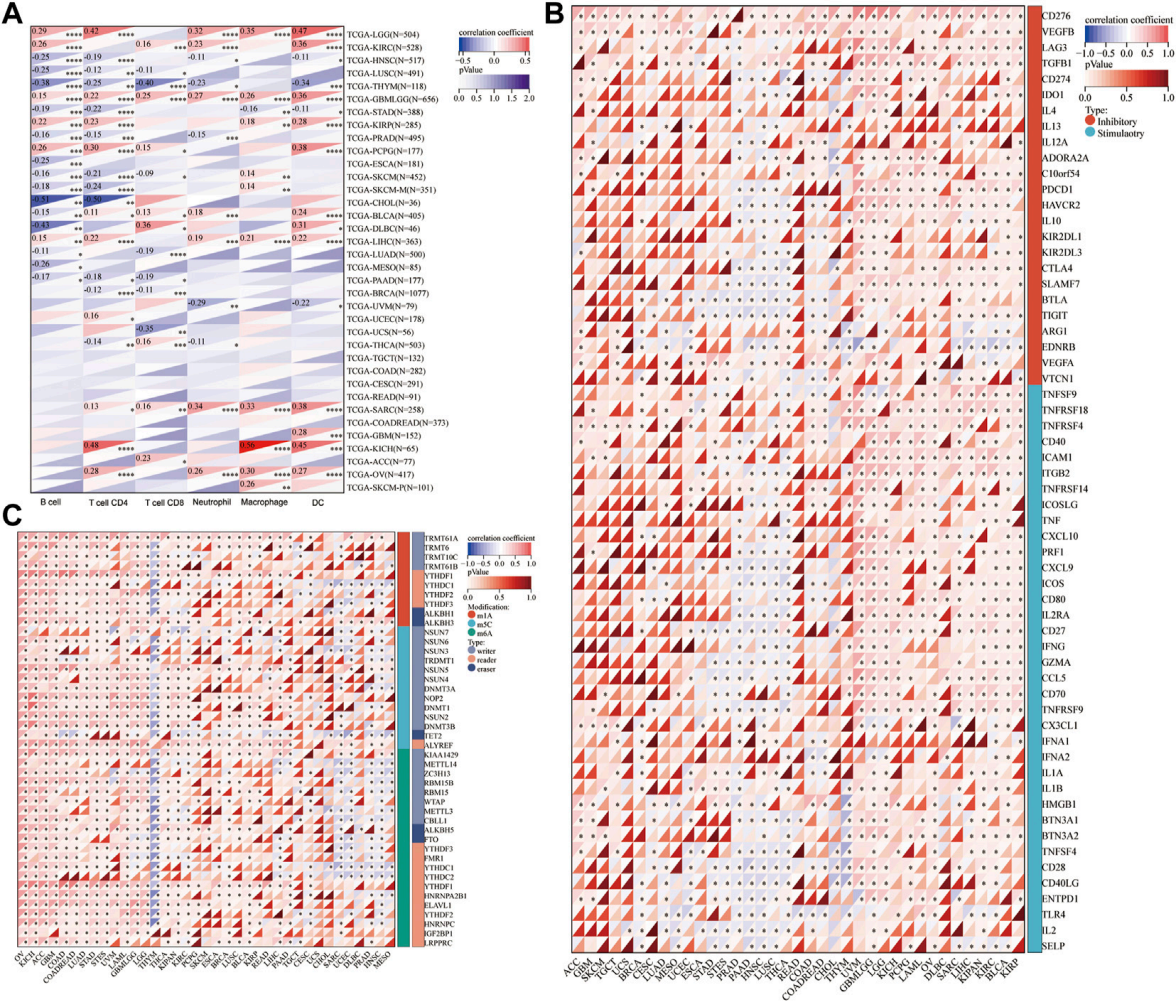
Correlation analysis for HM13 expression with immune cell infiltration levels, immune checkpoints, and RNA modification-related molecules. **(A)**. The correlation between HM13 expression and tumor infiltration levels was based on the TIMER database. **(B)**. Pan-cancer co-expression analysis for HM13 and immune checkpoint genes. **(C)**. Co-expression analysis for HM13 and RNA modification-related molecules. *p < 0.05, **p < 0.01, ***p < 0.001, ****p < 0.0001.

Existing literature confirms that the immune microenvironment is crucial for the development and prognosis of tumors. Previous studies show that HM13 is abnormally high expressed in several tumors, which is related to the prognosis of these patients. However, the relationship between HM13 expression and the immune microenvironment remains unclear. The TIMER database provides information on six types of immune cell invasion in 39 tumors. Thus, using the TIMER database, we analyzed the expression of HM13 and the levels of immune cell infiltration. The results suggested that HM13 was correlated significantly with B cells in several tumors, and other immune cells in LGG, KIRC, THYM, GBM, and LIHC (Figure 4A). Next, we obtained 60 immune checkpoint genes (24 inhibitory and 36 stimulatory) from relevant literature and analyzed the correlation between HM13 expression and immunosuppressive points (Thorsson et al., 2018). HM13 expression was correlated significantly with CD276, VEGFB, LAG3, TNFSF9, and TNFRSF18 (Figure 4B). Furthermore, HM13 showed a significant correlation with multiple immunosuppressive point genes implicated in LGG, UVM, LIHC, KIRC, and other tumors (Figure 4B). RNA methylation is reportedly involved in maintaining normal physiology, as well as, pathogenesis and development of diseases. We analyzed the correlation between HM13 expression and RNA modification-related molecules, including m1A, m5C, and m6A. A significant association of HM13 expression with RNA modification-related molecules in OV, KICH, ACC, GBM, LGG, and other tumors was observed (Figure 4C).

### Correlation between HM13 expression and genome heterogeneity

**FIGURE 5:**
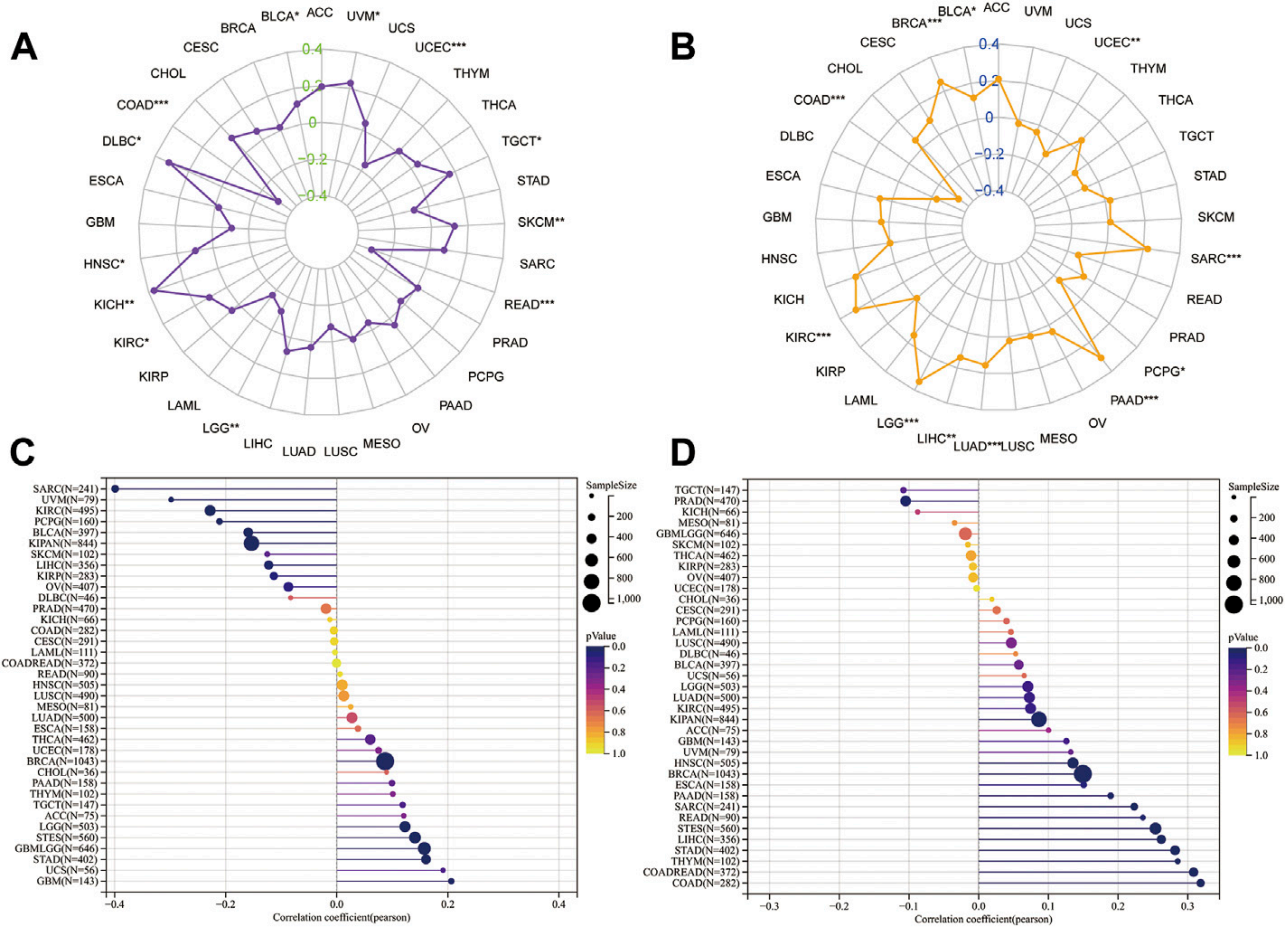
Correlation analysis for HM13 expression with MSI **(A)**, TMB **(B)**, purity **(C)**, and ploidy **(D)**. *p < 0.05, **p < 0.01, ***p < 0.001.

Genomic heterogeneity is an important molecular biomarker for several tumor types and has important clinical applicability. Thus, we examined the relationship between HM13 expression and MSI, TMB, purity, and ploidy. MSI analysis showed that HM13 expression correlated significantly with MSI of COAD, DLBC, KICH, LGG, READ, UCEC, and UVM (Figure 5A). TMB analysis showed that a significant correlation was present between HM13 expression with BRCA, COAD, KIRC, LGG, LIHC, LLUAD, PAAD, PCPG, SARC, and UCEC (Figure 5B). Purity analysis showed that HM13 expression also showed a marked association with the tumor purity in SARC, UVM, KIRC, GBM, UCS, and LGG (Figure 5C). Further, ploidy analysis showed that HM13 expression was correlated significantly with polyploidy in COAD, STAD, LIHC, STES, READ, and PAAD (Figure 5D).

### Drug sensitivity analysis

**FIGURE 6:**
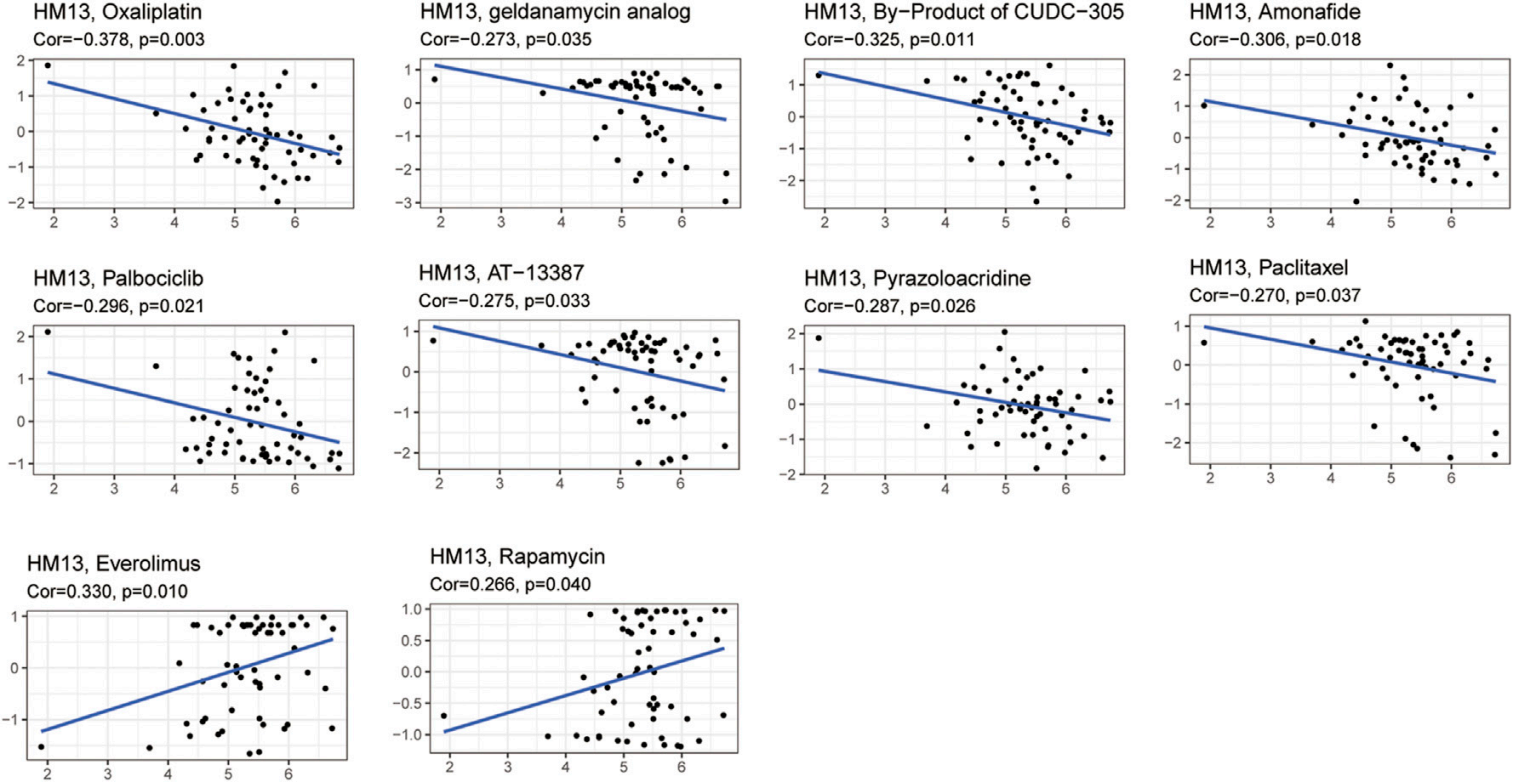
Relationship between HM13 expression and drug sensitivity.

Given the developments in precision and individualized medicine, the effects of gene expression patterns on drug sensitivity are increasingly being appreciated. Hence, we evaluated the relationship between the mRNA expression of HM13 and drug sensitivity. Based on the CellMiner database, 10 drugs associated with the expression of HM13 were identified. Among them, oxaliplatin (Cor = -0.378, *p* = 0.003), geldanamycin analog (Cor = -0.273, *p* = 0.035), B = by-product of CUDC-305 (Cor = -0.325, *p* = 0.011), amonafide (Cor = -0.306, *p* = 0.018), palbociclib (Cor = -0.296, *p* = 0.021), AT-13387 (Cor = -0.275, *p* = 0.033), pyrazoloacridine (Cor = -0.287, *p* = 0.026), and paclitaxel (Cor = -0.270, *p* = 0.037) showed a significant negative correlation with the expression of HM13 (Figure 6). However, a significant positive correlation was observed between sensitivity to everolimus (Cor = 0.330, *p* = 0.010) and rapamycin (Cor = 0.266, *p* = 0.040) and the expression of HM13 (Figure 6). Additionally, GDSC data showed that low HM13 expression has a lower IC50 value, including AICAR, AKT. Inhibitor, AMG.706, Axitinib, and AZD.0530 (Supplementary Figure S3). These results suggested that the expression of HM13 might affect the sensitivity of antitumor drugs.

### Diagnostic and prognostic value of HM13 in HCC

**FIGURE 7:**
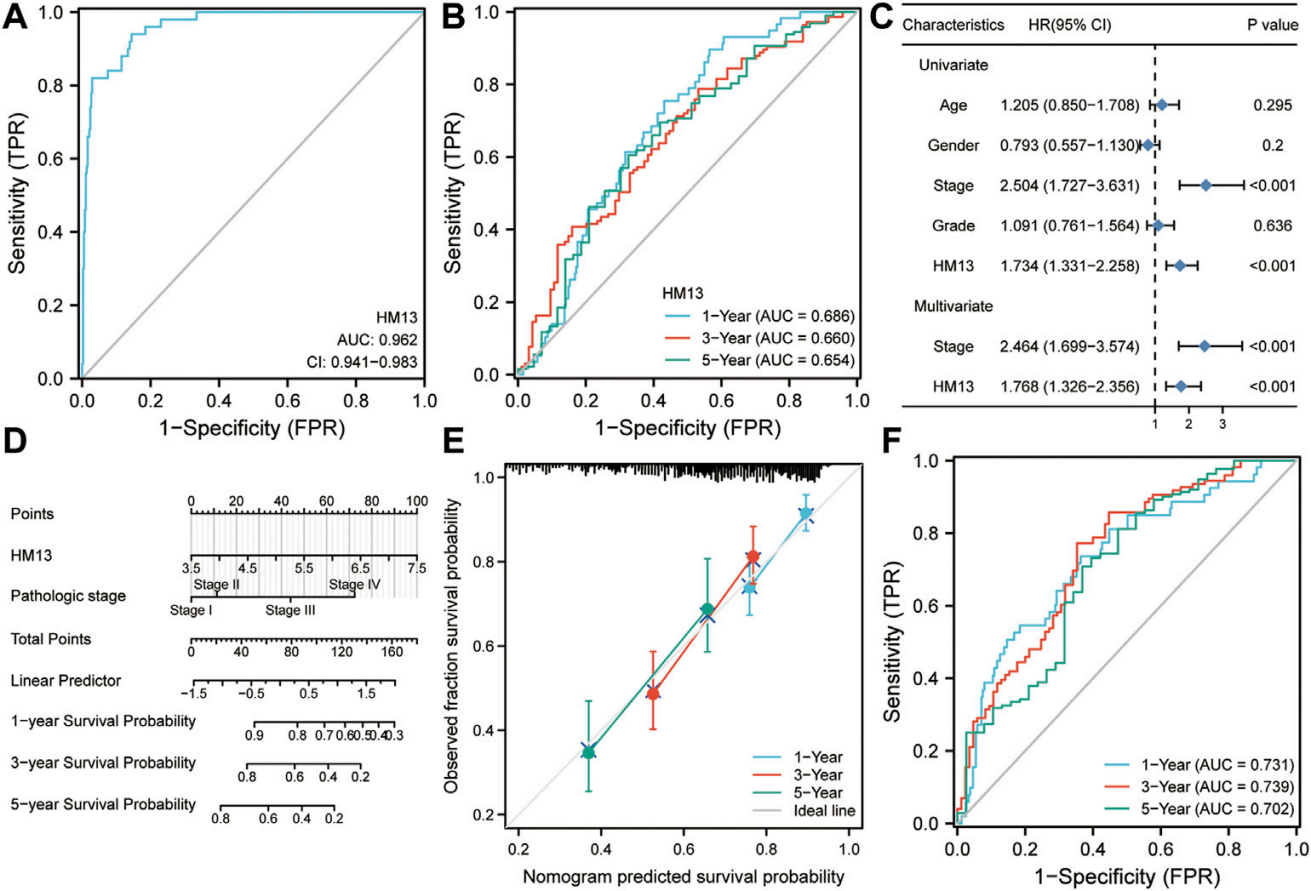
Diagnostic and prognostic value of HM13 expression in hepatocellular carcinoma. **(A)**. ROC analysis for HM13 in the diagnosis of hepatocellular carcinoma. **(B)**. ROC analysis for HM13 for predicting 1-, 3-, and 5-years overall survival in patients with hepatocellular carcinoma. **(C)**. Univariate and multivariate Cox regression analyses show that HM13 is an independent predictor of poor prognosis in hepatocellular carcinoma. **(D)**. The nomogram was constructed by combining pathological staging and HM13 expression. **(E)**. Calibration was used to evaluate the validity of the nomogram. **(F)**. ROC analysis was used to evaluate the predictive efficacy of the nomogram.

Based on the results of the above analyses, we investigated the role of HM13 in the occurrence and development of HCC. First, we evaluated the diagnostic value of HM13 expression in HCC. ROC analysis showed that the expression of HM13 could differentiate tumor tissues from normal tissues to a certain extent, and the AUC was 0.962 (Figure 7A). Subsequently, we evaluated the predictive efficacy of HM13 expression in the overall survival time of patients with HCC, and the AUCs for 1-, 3-, and 5-years were 0.686, 0.660, and 0.654, respectively (Figure 7B). In addition, we also found that HM13 expression was significantly higher in the high histologic grade and high AFP expression compared with low histologic grade and low AFP expression (Supplementary Figures S4A,B). Methylation analysis showed that significantly higher promoter methylation level of HM13 was observed in normal tissue (Supplementary Figure S4D). Genetic alteration displayed that 1.4% of HCC patients harbor HM13 amplification mutation (Supplementary Figure S4E). Moreover, we also examined the relationship between HM13 expression and the expression of EMT-associated genes (Supplementary Figure S4F). Next, the clinical data were integrated, and by univariate and multivariate Cox regression analyses, we confirmed that HM13 expression was an independent prognostic factor for HCC (Figure 7C). To promote the application of HM13 expression in the assessment of clinical prognosis, we constructed a nomogram in combination with the pathological staging (Figure 7D). Calibration analysis suggested a relatively stable performance of the nomogram for predicting the 1-, 3-, and 5-years overall survival rate of patients with HCC (Figure 7E). ROC analysis showed that the nomogram was robust with AUCs of 0.731, 0.739, and 0.702, respectively, for 1-, 3-, and 5-years overall survival rates of patients with HCC (Figure 7F).

**FIGURE 8:**
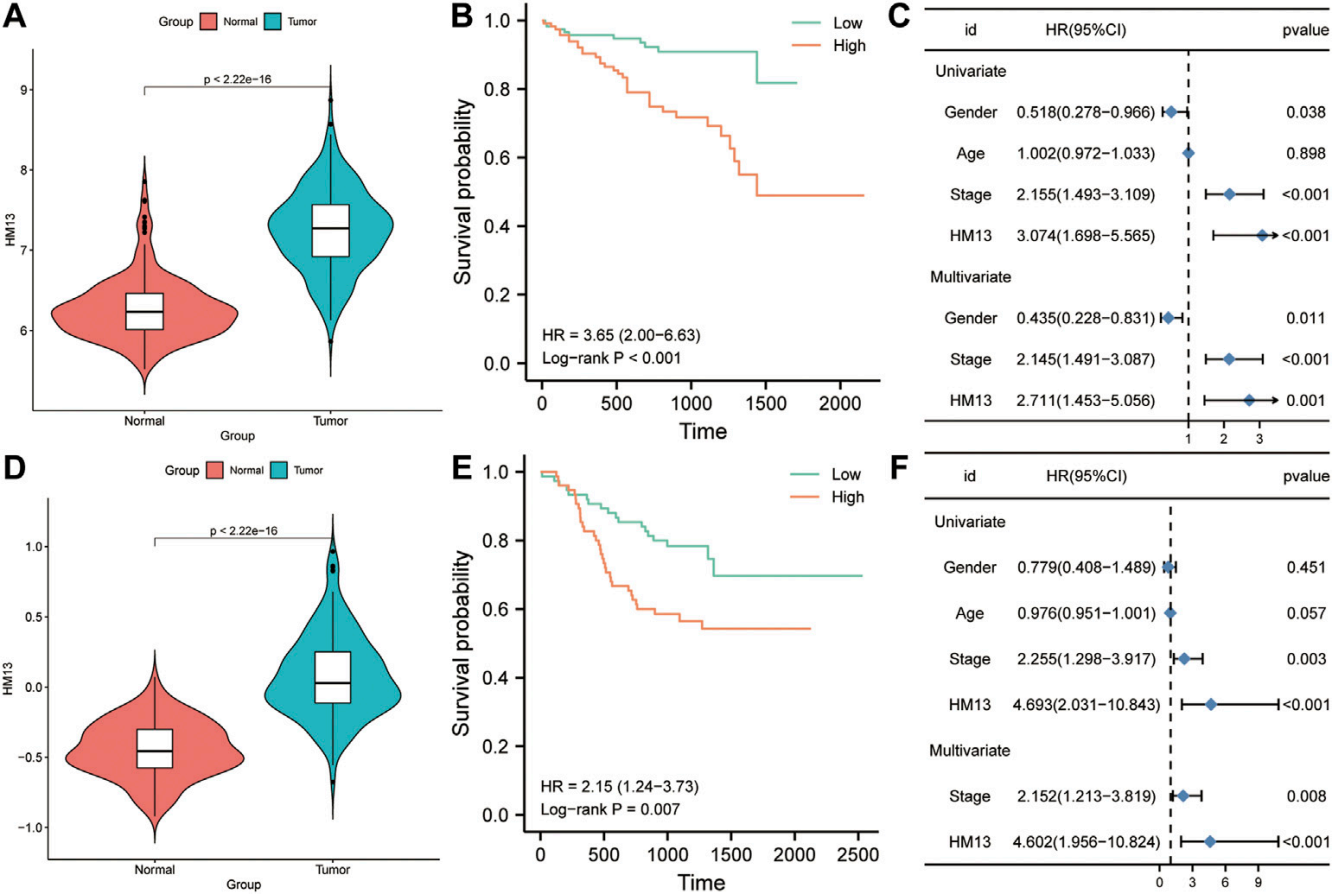
The expression level and clinical significance of HM13 in hepatocellular carcinoma (HCC). **(A)**. The mRNA expression of HM13 in ICGC cohort. **(B)**. Based on the ICGC cohort, Kaplan-Meier plot was used to evaluate the relationship between the expression of HM13 and overall survival. **(C)**. Univariate and multivariate COX analyses were carried out to determine the effect of HM13 on overall survival. **(D)**. The protein level of HM13 in CPTAC cohort. **(E)**. Based on the CPTAC cohort, Kaplan-Meier plot was used to evaluate the relationship between the expression of HM13 and overall survival. **(F)**. Univariate and multivariate COX analyses were carried out to determine the effect of HM13 on overall survival.

To further determine the expression and prognostic value of HM13 in HCC, RNA-seq data were obtained from the ICGC database and standardized processing was performed. Differential analysis indicated significant overexpression of HM13 in HCC tumor tissues (Figure 8A). In addition, the analysis from GEO datasets also indicated that HCC tissues highly expressed HM13, while it was poorly expressed in the non-tumor tissues (Supplementary Figures S5A–D). The Kaplan-Meier survival curve analysis demonstrated that patients with high HM13 expression had worse overall survival (Figure 8B). Results of univariate and multivariate Cox regression indicated that HM13 expression was independent of other clinical factors for HCC prognosis (Figure 8C). Additionally, Z-score normalized HCC protein expression profiles were obtained from the CPTAC database. Subsequently, we analyzed the protein expression of HM13 between normal and HCC tumor tissues, and significant HM13 overexpression was observed in HCC tumor tissues (Figure 8D). As well, a higher expression level of HM13 was observed in high tumor stages compared to low tumor stages (Supplementary Figure S4C). Kaplan-Meier survival curve also demonstrated that high HM13 expression was associated with a poor prognosis (Figure 8E). Furthermore, univariate and multivariate Cox regression analysis also indicated that HM13 expression at the protein level was an independent prognostic factor for HCC (Figure 8F).

**FIGURE 9:**
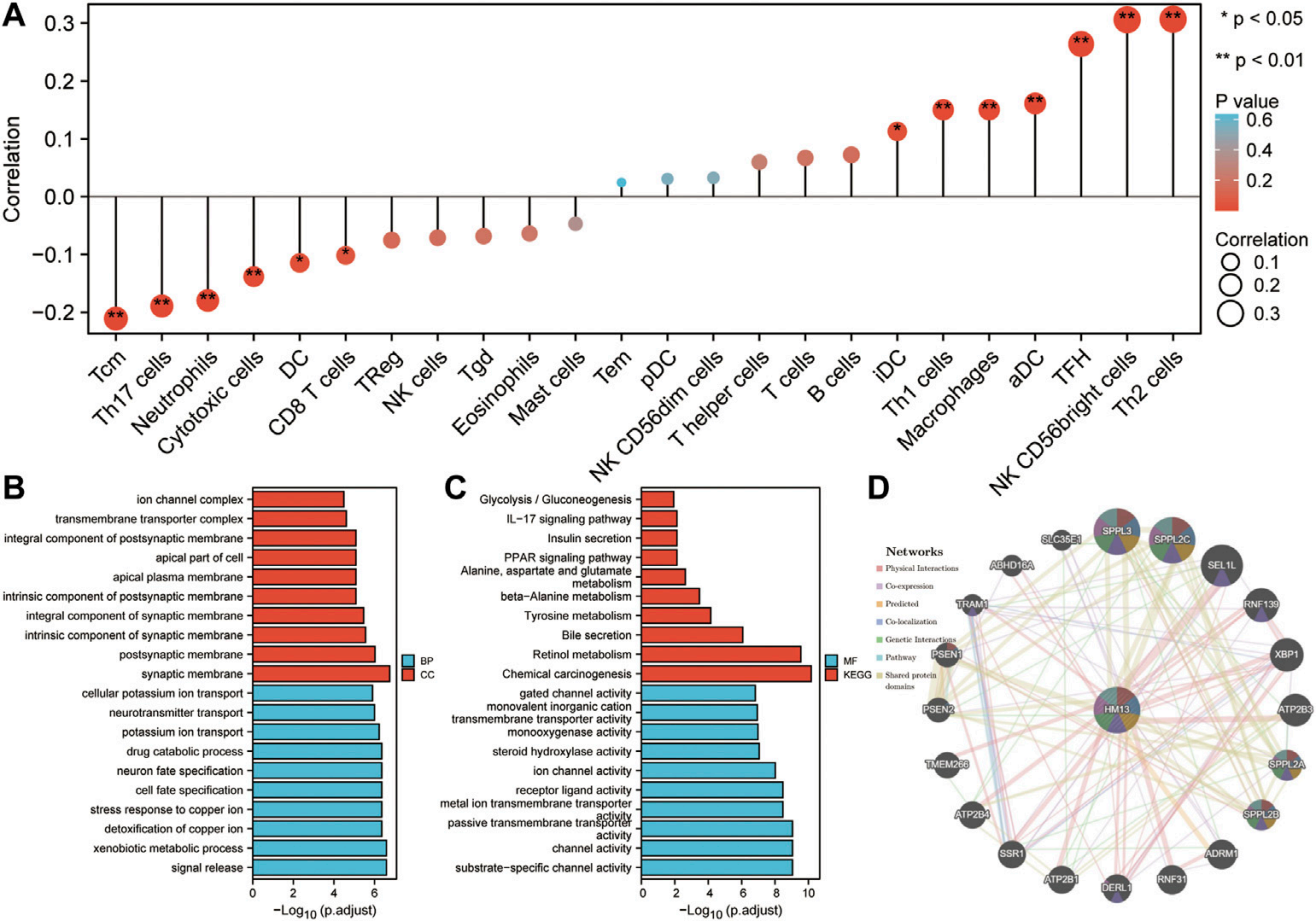
Potential function of HM13. **(A)**. Based on ssGSEA method, the relationship between HM13 expression and immune cell infiltration in hepatocellular carcinoma was evaluated. GO and KEGG functional enrichment analysis of the molecules interacted with HM13 **(B,C)**. **(D)**. Protein-protein interaction network of HM13 was constructed based on the comPPI.

Based on TCGA RNA-seq data and the ssGSEA algorithm, we evaluated the infiltration levels of 24 kinds of immune cells in HCC and calculated their correlation with HM13 expression. HM13 expression showed a strong positive correlation with TH2 cells, NK CD56 cells, and TFH cells, while a negative correlation was observed with Tcm and Th17 cells (Figure 9A). To elucidate the potential function of HM13 expression in HCC, we classified the samples into high- and low-risk groups based on the median expression of HM13, the differential genes between the two groups were obtained, and GO and KEGG enrichment analyses were performed. Results of enrichment analysis showed that HM13 was mainly associated with cellular potassium ion transport, glycolysis/gluconeogenesis, IL-17 signaling pathway, and the PPAR signaling pathway (Figures 9B,C). Subsequently, we identified potential interaction of HM13 using GeneMAN. The results suggested the potential interaction of HM13 with SPPL3, SPPL2C, SPPL2A, and SPPL2B (Figure 9D).

### HM13 was associated with the proliferation, migration and invasion of HCC cells

**FIGURE 10:**
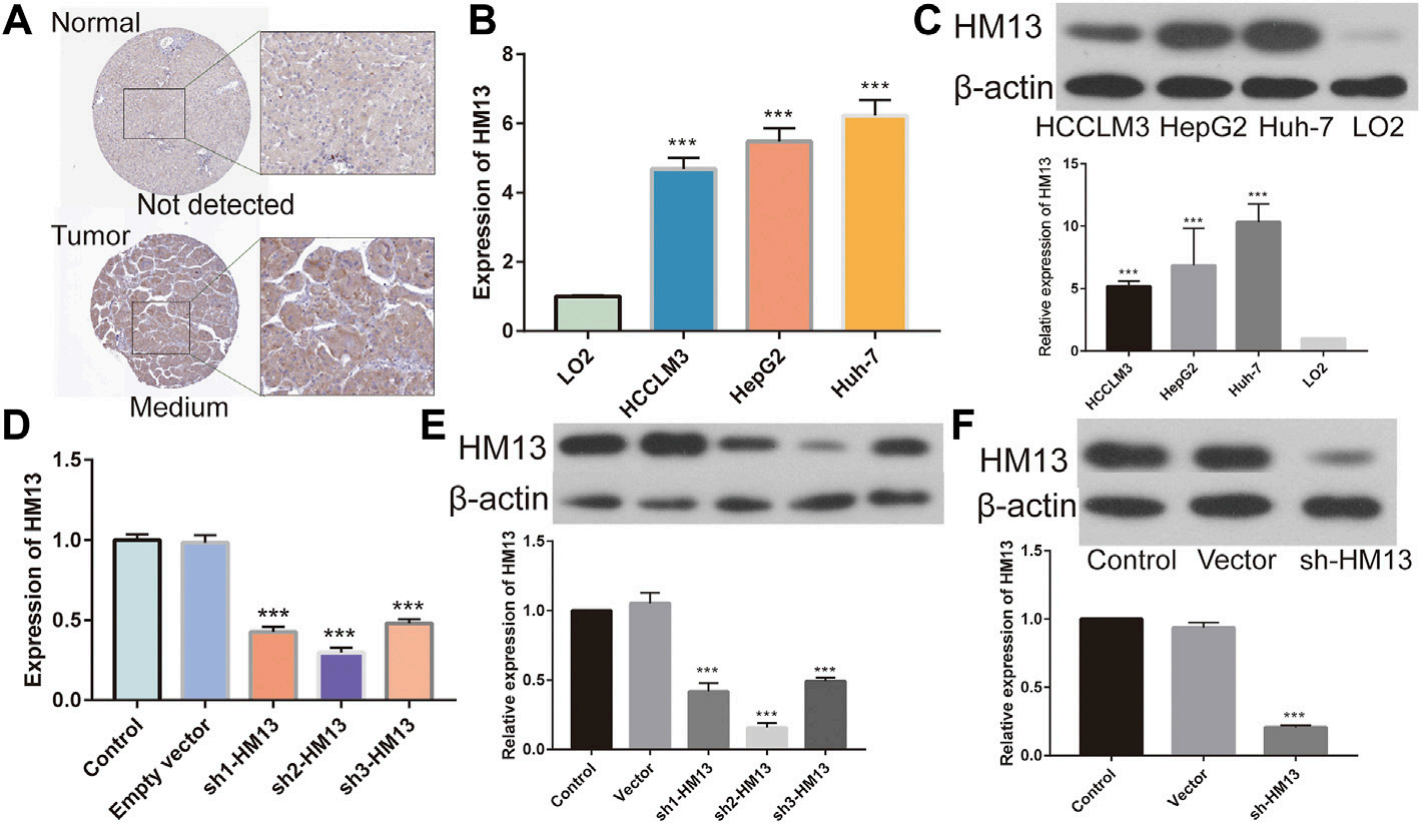
Validation of HM13 in HCC cell lines. **(A)**. Based on the human protein atlas (HPA) database, immunohistochemistry validated that the protein level of HM13 in HCC. **(B)**. Real time-qPCR was used to determine the mRNA expression of HM13 in HCC cell lines. **(C)**. Western blot was applied to evaluate the protein level of HM13 in HCC cell lines. The knock down efficiency was determined by real time-qPCR **(D)** and western blot **(E)** in Huh-7 cells. **(F)**. Knock down efficiencies of SH-HM13 was confirmed by western blot in Huh-7 cells. Experiments were repeated three times; *p < 0.05, **p < 0.01, ***p < 0.001.

**FIGURE 11:**
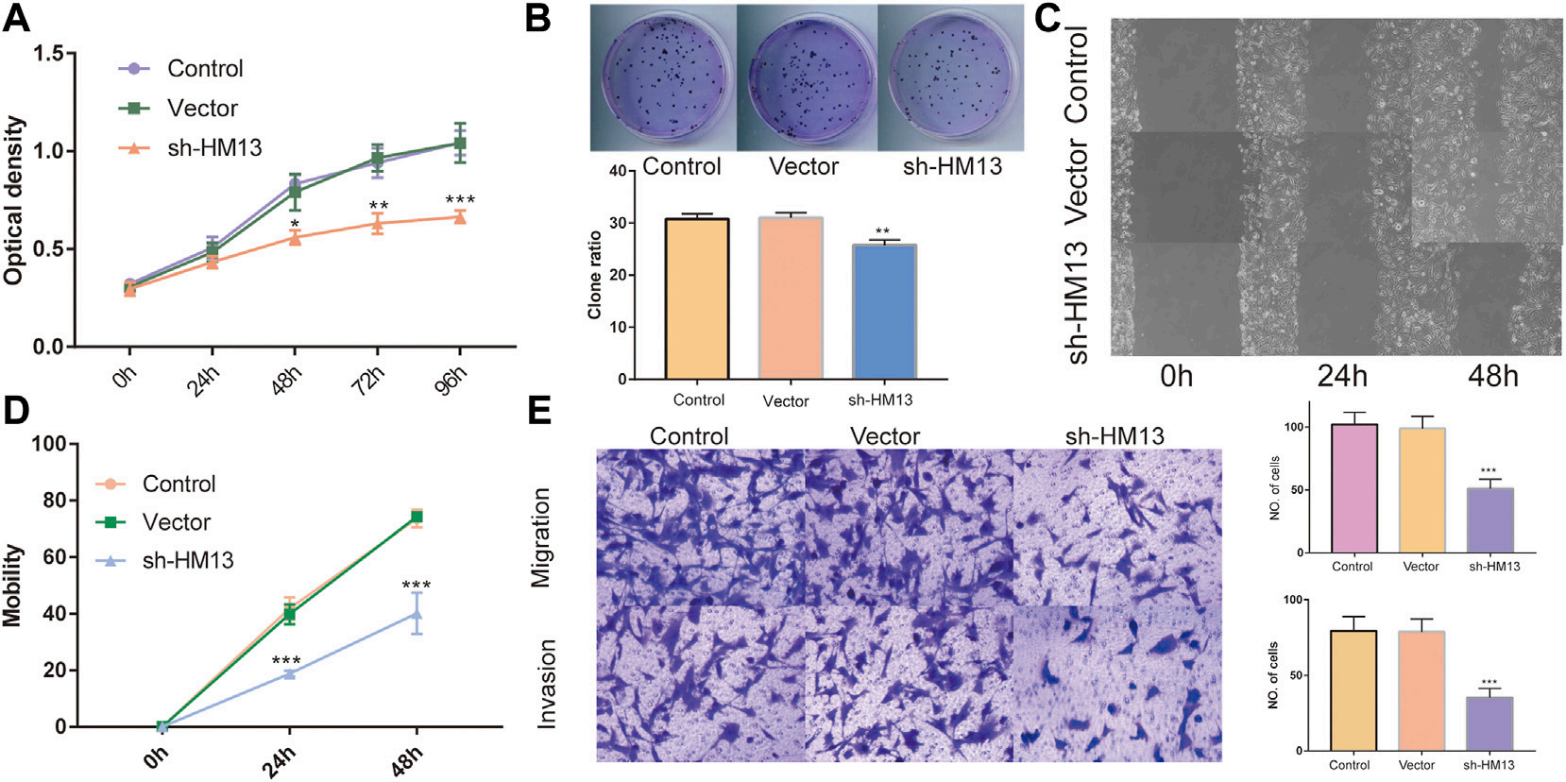
The effect of HM13 knockdown on Huh-7 cells functions. Huh-7 cell proliferation was measured by CCK8 assay **(A)** and colony formation **(B)**. **(C,D)**. Cell migration was determined by scratch wound assay. **(E)**. Cell migration and invasion were determined by transwells assay. Experiments were repeated three times; *p < 0.05, **p < 0.01, ***p < 0.001.

**FIGURE 12:**
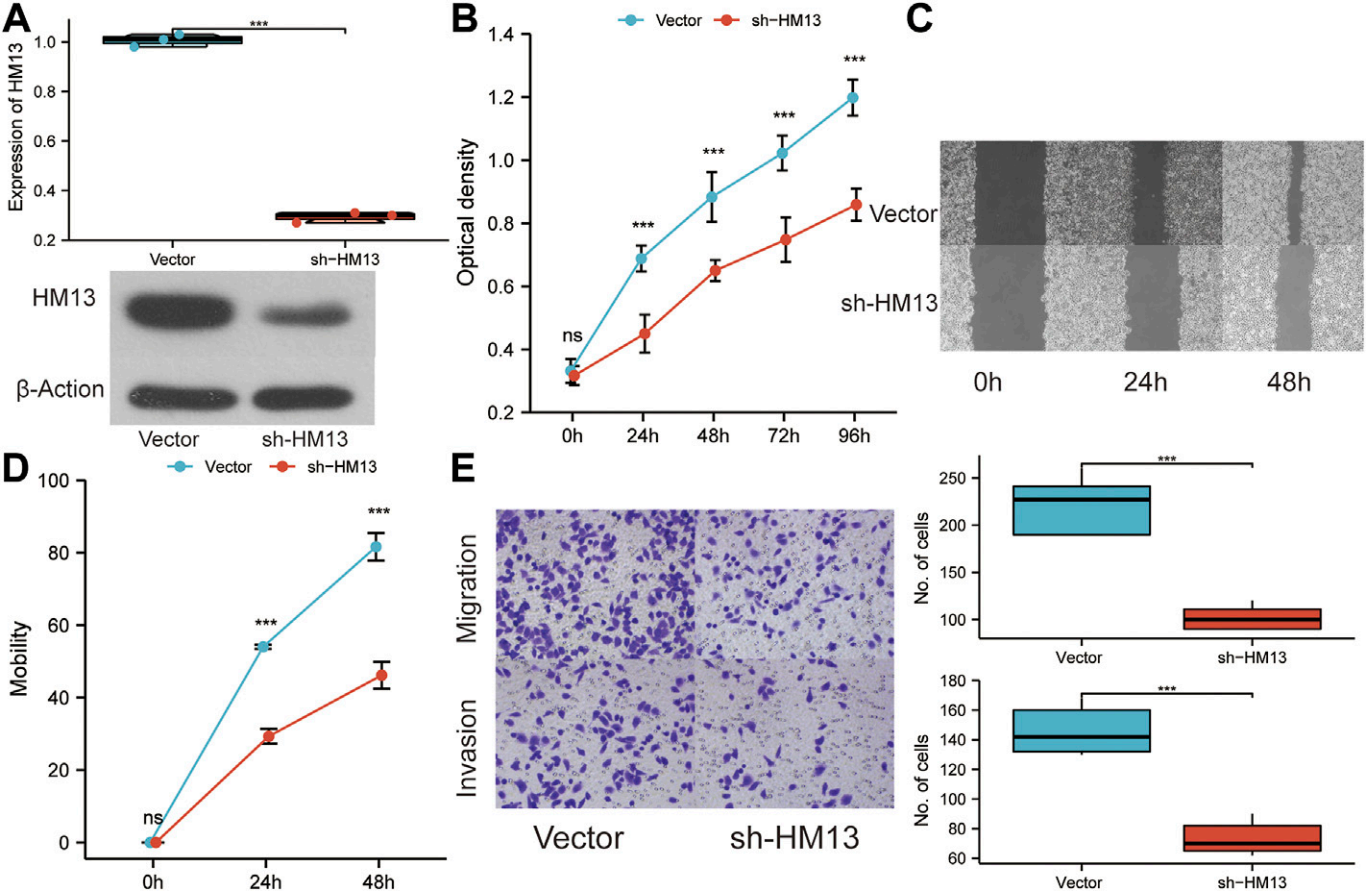
The effect of HM13 knockdown on HCCLM3 cell function. **(A)**. RT-PCR and western blot were used to validate the knockdown of HM13 expression. **(B)**. Using the CCK8 assay, HCCLM3 cells were evaluated for their proliferative capacity. **(C)**. **(D)**. Scratch assays were used to examine cell migration abilities. **(E)**. Cell migration and invasion were assessed in HCCLM3 cells using the transwell assay. Experiments were repeated three times; ***p < 0.001.

The immunohistochemical data for HM13 expression in HCC tissues were extracted from the HPA database (https://www.proteinatlas.org/). The results showed a higher HM13 expression in HCC tumor tissues (Figure 10A). Subsequently, we further investigated the mRNA and protein level expression of HM13 in HCC cell lines. The results showed higher mRNA and protein levels of HM13 expression in HCC lines, including HCCLM3, Huh-7, and HepG2, as compared to the normal liver cell line, LO2 (Figures 10B,C). A total of three shRNA constructs of HM13, namely sh1-HM13, sh2-HM13, and sh3-HM13 were verified for their interference efficiency in Huh-7 cells. The results showed that sh2-HM13 significantly interfered with the mRNA and protein expression of HM13 (Figures 10D,E) and was subsequently selected for further experiments; it was named sh-HM13 (Figure 10F). CCK8 assays proved that interfering HM13 expression could significantly inhibit the proliferation of Huh-7 cells (Figure 11A). Assays of clone formation showed that the knockdown HM13 inhibited the capacity of Huh-7 cells to form colonies (Figure 11B). The results of the scratch assay suggested that interfering with HM13 expression could significantly lead to inhibited cell migration ability (Figures 11C,D). Further, results of the transwell migration and invasion assay confirmed that interfering with HM13 expression could significantly suppress cell migration and invasion abilities (Figure 11E). Moreover, we knocked down HM13 in HCCLM3 cells (Figure 12A). The results of CCK8 assay revealed that suppression of HM13 significantly reduced cell viability (Figure 12B). Additionally, the scratch test and transwell experiments demonstrated that knockdown HM13 inhibited metastasis and invasion of HCCLM3 cells substantially (Figures 12C–E).

## Discussion

Cancer is the leading threat to human health because of its complicated pathogenesis, rapid progression, and lack of effective treatment. Understanding high-risk factors for cancer, early detection, and effective treatment are prerequisites to improve the overall survival and prognoses of these patients (Roy et al., 2021). Pan-cancer analysis can help reveal the common basis underlying the development and occurrence of different tumors, and provide new clues for elucidating the mechanism of cancer occurrence and developing personalized precision therapy (Korenjak and Zavadil, 2019). At present, researchers are focusing on pan-cancer genomic analyses and correlating these findings with the results of multi-omics to identify new tumor markers and therapeutic targets (ITP-CAOWG Consortium, 2020). Herein, we comprehensively discussed the expression of HM13 in several tumors for the first time based on RNA-seq data from TCGA. HM13 expression was abnormal and up-regulated in several tumor tissues. Combining pairing analysis with the GETx database, we further confirmed enhanced HM13 expression in UCEC, CHOL, BRCA, KIRC, HNSC, LIHC, KIRP, LUSC, LUAD, BLCA, COAD, ESCA, PRAD, STAD, and READ tumor tissues as compared to those adjacent to the corresponding carcinoma. Through univariate Cox regression and Kaplan-Meier survival analyses, we confirmed high HM13 expression in ACC, KIRP, UVM, LIHC, LGG, HNSC, and KIRC. Abnormal HM13 expression was associated with worse progression-free survival and overall survival.

The role of the tumor immune microenvironment in tumor development and prognosis has attracted increasing attention from researchers (Binnewies et al., 2018). Herein, immune cells can interact with cancer cells to promote/inhibit the growth and metastasis of tumors (Locy et al., 2018). While advances have been made in tumor immunotherapy in clinical settings, due to the complicated relationship between the immune microenvironment and tumor cells, its efficiency needs further improvement (Bader et al., 2020; Zhang and Zhang, 2020). In the present study, HM13 expression correlated negatively with T- and B cell infiltration in a variety of tumors. A positive correlation was observed with macrophages in LGG, LIHC, SARC, KICH, OV, and SKCM. Furthermore, the ssGSEA algorithm verified that HM13 expression showed a certain positive correlation with macrophages and a negative correlation with CD8T cells in HCC. Macrophages secrete pro-inflammatory mediators to promote tumor cell proliferation and help tumor cell migration through paracrine routes (Ruffell and Coussens, 2015; Pathria et al., 2019). CD8T cells are important tumor killer cells and can inhibit their growth (Farhood et al., 2019; Raskov et al., 2021). Through immune checkpoint gene expression analysis, the expression of HM13 was found to exhibit a significant correlation with several tumor inhibitory genes, especially VEGFB, LAG3, CD274, and TGFB1. Accumulating evidence suggests that abnormally high expression of these genes is associated with the poor prognosis of cancer patients (Andrews et al., 2017; Lacal and Graziani, 2018; Chen et al., 2019; Cane et al., 2021). These results can explain why HCC patients with high HM13 expression are likely to have a poor prognosis. Therefore, we reasonably speculated that the expression of HM13 could promote the interaction between tumor and immune cells, which provides a new indicator for monitoring immunotherapy or a new adjuvant therapeutic target. Previous studies suggest that gene expression correlates with drug sensitivity (Vuong et al., 2014; Salvadores et al., 2020). In this study, we found that the expression of HM13 correlated negatively with the sensitivity of several drugs. This suggested that HM13 mRNA expression could predict drug responses, thus highlighting the potential of the HM13 as a drug target.

The protein encoded by the HM13 gene is a signal peptide peptidase, which is mainly localized to the endoplasmic reticulum. SPP is mainly involved in protein hydrolysis, especially by mediating the intramembrane cleavage of type 2 transmembrane proteins, which plays an important role in maintaining protein homeostasis (Mentrup et al., 2020). Abnormal expression of HM13 is implicated in several diseases. Hsu FF et al. showed that SPP levels are abnormally high in lung cancer and breast cancer, and it may interact with FKBP8 to regulate the proliferation and migration/invasion ability of lung tumor cells (Hsu et al., 2019). Wei JW et al. show that HM13 is significantly upregulated in high-grade gliomas, and its expression correlates positively with the degree of malignancy (Wei et al., 2017). Knocking out HM13 significantly inhibits tumor cell survival, reduces cytokine secretion in EGFRvIII glioma cells, and affects the biological behavior of surrounding cells by mediating the TGF-β pathway (Delman, 2020). In addition, SPP is associated with the maturation of hepatitis C virus core protein and may play a role in HCC (Moriishi, 2017). In this study, we confirmed the trend of abnormally high HM13 expression in HCC tissues in multiple databases, both at mRNA and protein levels. It was an independent risk factor for poor prognosis in HCC. We also verified the higher expression of HM13 in HCC cell lines. Moreover, to further examine the function of HM13 in the progression of HCC, we constructed shRNA plasmids for HM13. Interfering with HM13 expression significantly inhibited the growth and migration/invasion of HCC cells. These results suggested that HM13 may play an important role in tumor genesis and development, thus making it a promising new marker or therapeutic target. In addition, the enrichment analysis showed that HM13 may act as effective parameters in regulating tumor metabolism. The GeneMANA displayed that HM13 may interact with the SPP-like proteases. Studies have shown that abnormal overexpression of SPP can activate Notch and mTORC signaling pathways and promote tumor proliferation and metastasis (Papadopoulou and Fluhrer, 2020). This suggested that HM13 might involve in the regulation of Notch and mTORC pathways.

However, there were still some limitations to be stated in the present study. We explored the relationship between HM13 expression and immune cell infiltration and the expression of immune checkpoint genes based on bioinformatics analysis. Further *in vivo* and *in vitro* experiments are important to confirm the significance of observations in this study.

In conclusion, we delineated the pan-carcinoma expression profile of HM13 and found that its abnormally upregulated expression correlated with poor prognosis in patients with tumors. The abnormal expression was significantly associated with the levels of immune checkpoint genes and immune cell infiltration. Furthermore, interference with HM13 expression in Huh-7 and HCCLM3 cells significantly suppressed proliferation and migration/invasion of tumor cells. Therefore, our findings suggested that HM13 was crucial for tumor genesis and development, and could be used as a marker for tumor diagnosis and prognostic evaluation.

## Data Availability

The datasets presented in this study can be found in online repositories. The names of the repository/repositories and accession number(s) can be found in the article/Supplementary Material.
