# Supplementary figures and images for: Pan-cancer analysis suggests histocompatibility minor 13 is an unfavorable prognostic biomarker promoting cell proliferation, migration, and invasion in hepatocellular carcinoma
Source: Front Pharmacol. 2022 Aug 15;13:950156. doi: 10.3389/fphar.2022.950156 (PMC9421072; doi:10.3389/fphar.2022.950156)

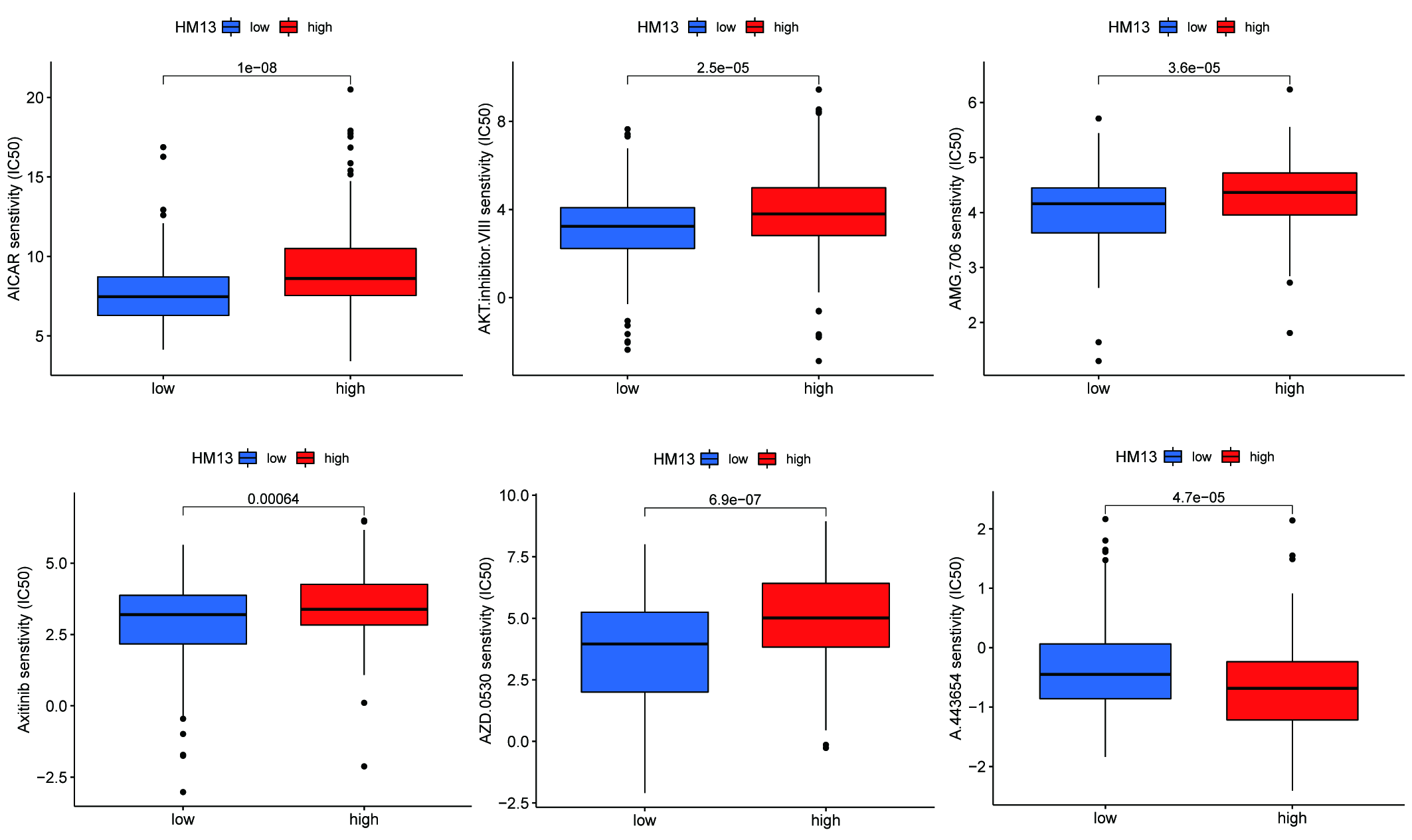

Supplement: Supplementary file 1 [file Image3.TIF]

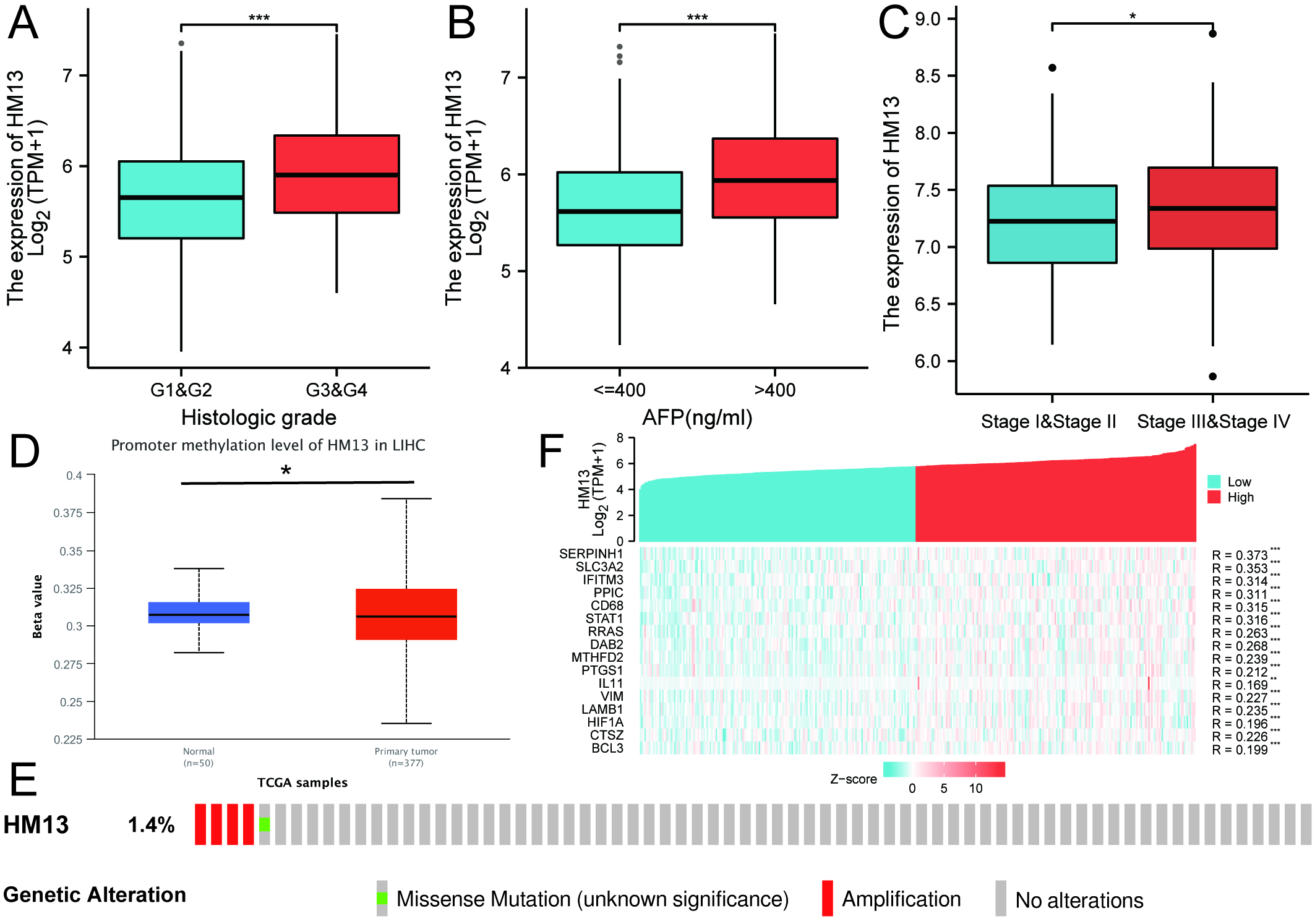

Supplement: Supplementary file 2 [file Image4.TIF]

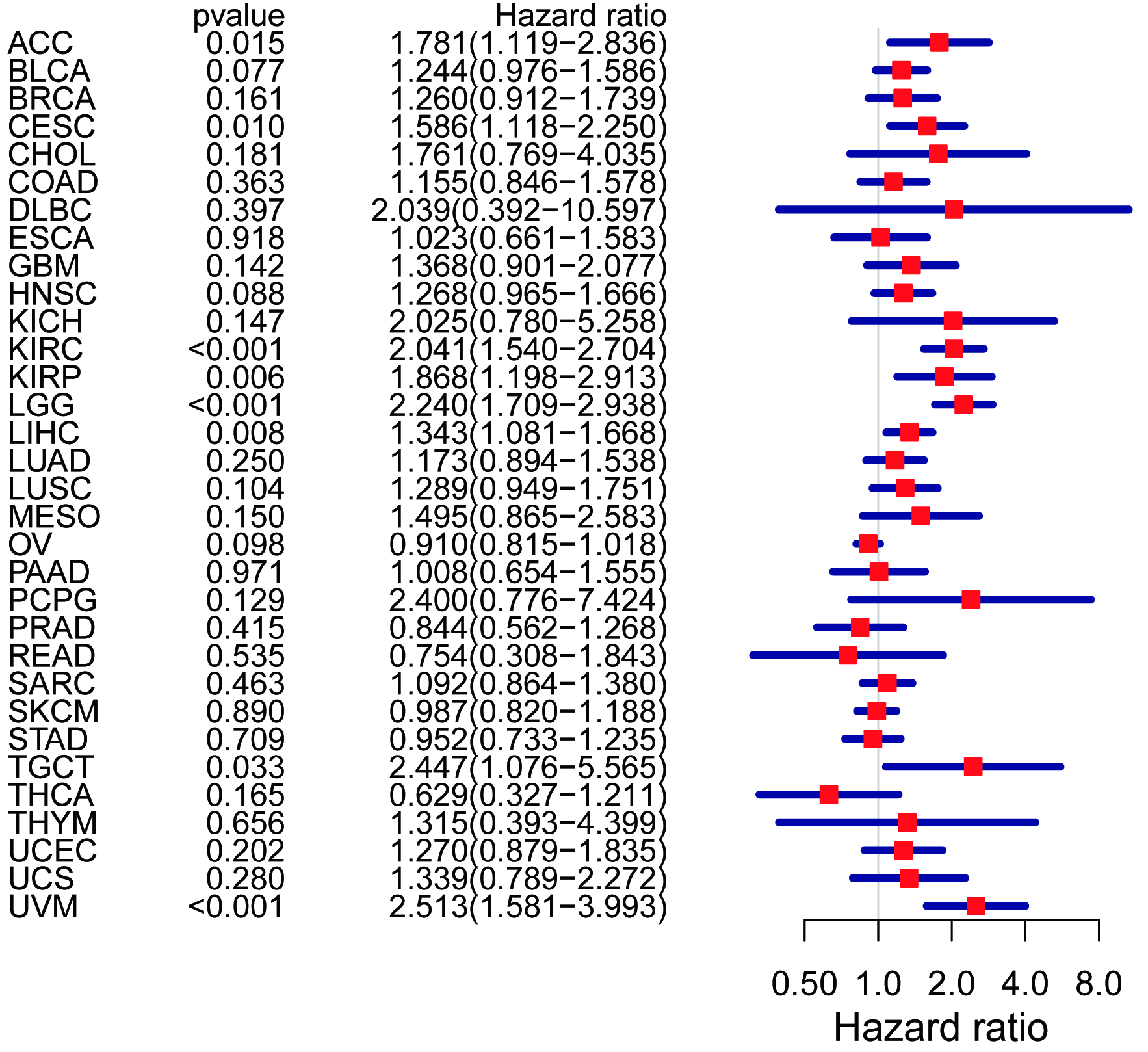

Supplement: Supplementary file 3 [file Image2.TIF]

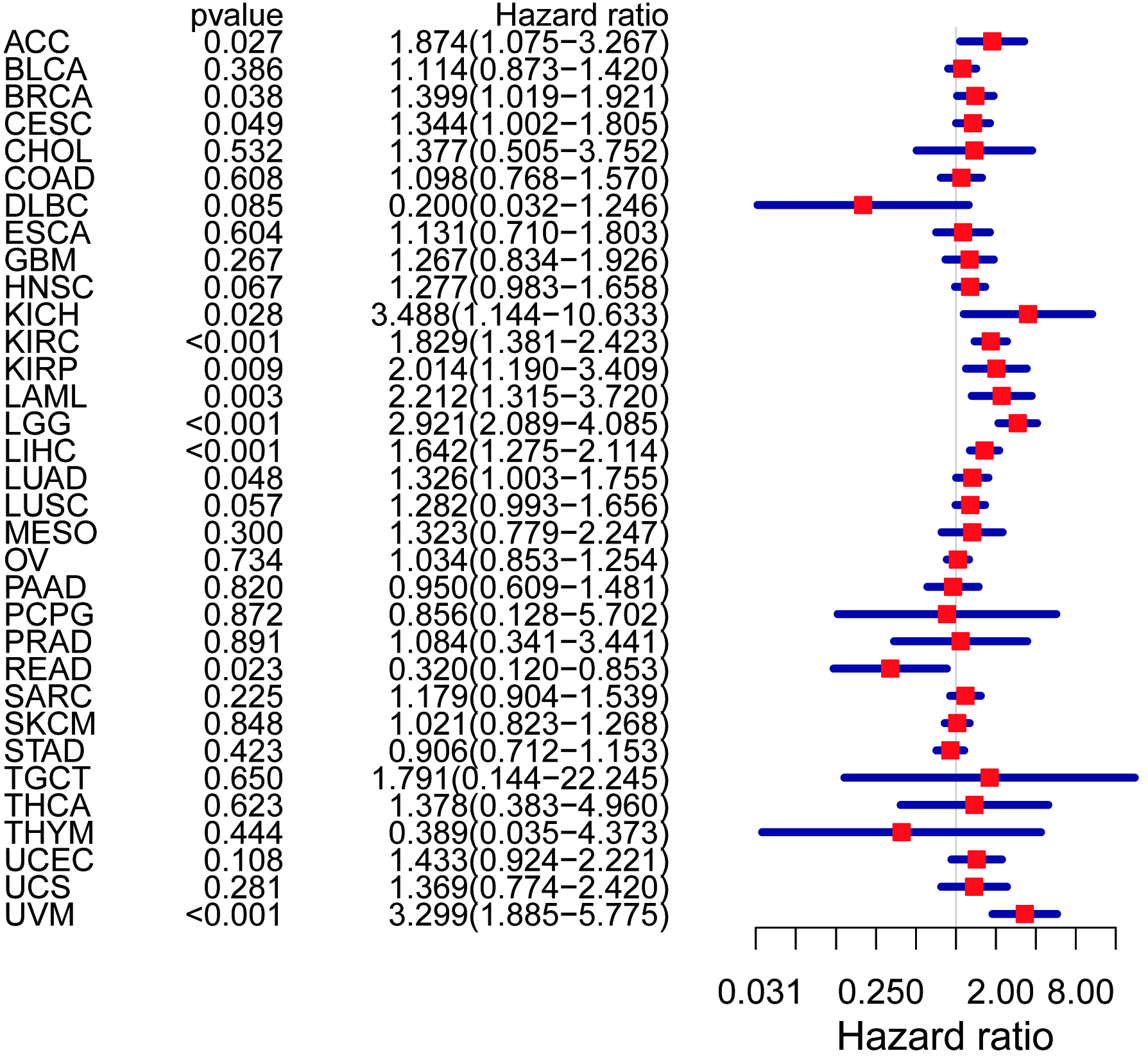

Supplement: Supplementary file 4 [file Image1.TIF]

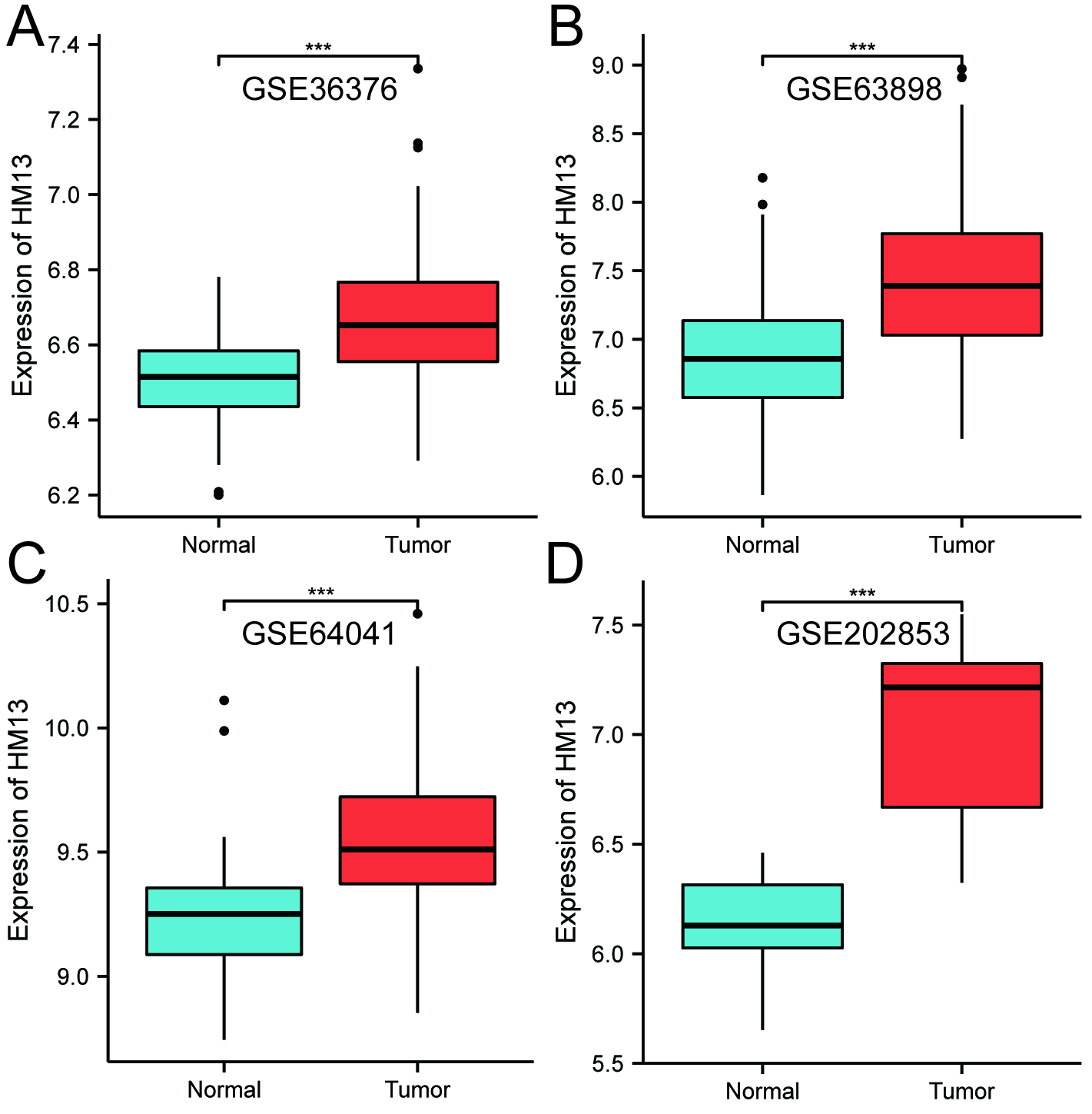

Supplement: Supplementary file 5 [file Image5.TIF]
